# Gender-affirming health care needs, barriers to care, and health and wellbeing in a broad nationwide sample of transgender people in Norway

**DOI:** 10.1186/s12889-025-25243-1

**Published:** 2025-12-05

**Authors:** Silje-Håvard Bolstad, Norman Anderssen, Bjørge H. Hansen, Ilan H. Meyer

**Affiliations:** 1 University of Agder, Kristiansand, Norway; 2 University of Bergen, Bergen, Norway; 3 University of California, Los Angeles, CA USA

**Keywords:** Transgender, Nonbinary, Gender incongruence, Gender-affirming, Health care, Barriers, Health

## Abstract

**Background:**

Transgender people report increased prevalence of mental distress and suicide attempts compared to the general population. Gender-based minority stressors such as discrimination and victimization are positively associated with mental health problems while social and medical gender transition is inversely associated with mental health problems. Barriers to accessing gender-affirming health care is reported by transgender people in many countries, including Norway which has a state-funded public health care system. The aim of the current study was to examine gender-affirming health care needs, barriers to care and health and wellbeing in a broad nationwide sample of transgender people in Norway.

**Methods:**

A nationwide sample with 579 transgender participants completed an anonymous online survey during June-September 2023. Chi-square tests and ANOVA were used to examine differences between gender identity groups.

**Results:**

Having obtained gender-affirming health care was more common among trans men and trans women as compared to nonbinary people. Participants generally reported being satisfied with the outcomes of gender-affirming hormonal and surgical treatments. Having obtained treatment entirely through private funding was reported by 32.5% of those using hormones and 49.5% of those who had obtained surgery. Not being able to afford hormones or surgery was reported by 45.9% and 65.5% among those with unmet treatment needs. Compared to trans men and trans women with unmet needs, nonbinary people with unmet needs were less likely to be under assessment or on a waiting list to obtain treatments. Of the total sample, 36.7% reported suicide attempts, 74.8% reported mental distress above clinical cut-off and 12.5% reported being satisfied with life.

**Conclusions:**

Transgender people in Norway reported high levels of mental distress and suicidality. Despite being young and with low income, a large proportion had obtained gender-affirming medical treatments entirely through private funding. Transgender people in need of gender-affirming health care could benefit from increased access to care through the state-funded public health care system in Norway.

**Supplementary Information:**

The online version contains supplementary material available at 10.1186/s12889-025-25243-1.

## Background

Transgender people are individuals whose gender identity differs from their sex assigned at birth. This population includes people with diverse gender identities including trans men, trans women, and nonbinary people who identify as neither man nor woman, or as both, or in other ways that depart from the traditional gender binary [1, 2]. Recent population estimates based on studies in the U.S., Sweden, The Netherlands, Belgium, Taiwan and New Zealand vary from 0.3% to 4.5% among adults and from 1.2% to 8.4% among children and adolescents [3].

Research from various countries has documented mental health disparities in young [4], adult [5–7], older [8] as well as ethnic minority [9] transgender populations as compared to the general population. Studies find reduced quality of life [10] and increased prevalence of depression [11], anxiety [12] and suicide attempts [13–15] among transgender people. Furthermore, studies from many countries have shown that transgender people face prejudice [16–18]. Transgender people are more often victims of violence and other discrimination and harassment behaviors [6, 19–22]. The minority stress model suggests that prejudice towards transgender people causes health disparities [23, 24]. Gender-based minority stress such as victimization, family rejection, internalized transphobia and gender identity non-disclosure is positively associated with mental health problems [25–29].

Gender transition refers to the process of making changes, to express and affirm one’s gender identity rather than the sex assigned at birth. Specific transition milestones are common to many transgender people, such as first identifying as a gender other than the sex assigned at birth, telling others about their transgender status, living part- or full time in line with their gender identity, and seeking out transition-related health care such as counselling, hormonal treatment or surgical procedures [30–32]. Completing social and medical transition steps is inversely associated with mental health problems [33–36]. Gender-affirming hormone treatment is associated with improved quality of life and reduced mental health problems such as depression and anxiety [37–43]. Gender-affirming surgeries are associated with improved quality of life and reduced psychological distress and suicidality [44–47], and patients are generally satisfied with surgical outcomes [48–52]. Common limitations of the existing research are small sample sizes, recruitment only in clinical settings, samples based on narrow diagnostic criteria, and not differentiating between gender identity groups [53].

Transgender people in many countries experience barriers to accessing health care [54, 55], including primary health [56], mental health [57–59], and gender-affirming health care [60–65]. Barriers include financial burden when gender-affirming care is not covered by health insurance policies and policies of state-funded health care systems [66–68], lack of competency in transgender-specific issues among clinicians [69–71], long waiting times or assessment periods [72, 73], discrimination in health care settings [74, 75], and transgender people’s healthcare avoidance based on lived or anticipated discrimination [76, 77]. Furthermore, nonbinary people may experience additional barriers such as pressure to conform to a binary gender identity, lack of available information on gender-affirming treatment options specifically for nonbinary people, and insurance policies not covering gender-affirming care for nonbinary people [78, 79].

### The Norwegian context

In Norway, transgender people are included in the Equality and Anti-Discrimination Act [80], which aims to “promote equality and prevent discrimination on the basis of gender, pregnancy. disability, sexual orientation, gender identity, gender expression or age”. Compared to other countries, Norway scores high on indexes of legal rights and public attitudes towards transgender and LGBTQ + people [81, 82]. Yet, 12–18% of the general population in Norway report negative attitudes toward transgender people [18]. In a nationwide study from 2020, 20–27% of transgender people had experienced discrimination or harassment over a year because of their gender identity, and 26–30% were victims of violence over five years, compared to 9% of cisgender people [6]. Compared to cisgender students, binary and nonbinary transgender students have increased odds of poor life satisfaction (3.78 and 3.12), mental health problems (2.48 and 4.07) and lifetime suicide attempt (5.56 and 6.12) [13].

Norway has universal health coverage [83]. Although private clinics are available, residents generally do not need to privately fund medically necessary health care, such as consultations, expensive prescription drugs and surgery. Publicly funded gender-affirming medical treatment is currently provided through one national clinic, which is divided into a section for adults and a section for children and youth. The clinic usually requires an assessment period of minimum one year, often including a psychiatric evaluation, and patients are expected to live in line with their gender identity in all life contexts throughout the assessment period [84]. Publicly funded hormone treatment, breast surgery, genital surgery, puberty blockers, permanent hair removal and voice training can be obtained by patients found eligible by the national clinic. In 2022, the section for adults received 647 referrals and started hormone treatment for 216 patients [85]. Recently established regional centers for gender incongruence provide non-medical care such as counselling.

Qualitative studies have documented that transgender people report extensive barriers to accessing gender-affirming medical treatments through the public health care system in Norway [61, 71, 86, 87]. In a study from 2023 with 24 interviews and a focus group, informants reported that they experienced the health care system to lack a general recognition of gender diversity, rejecting nonbinary people seeking gender-affirming care, and providing gender-affirming care based on a predefined transition pathway instead of individual treatment needs [61]. Some informants had sought private clinics despite having low income, and some could not afford private funding of their gender-affirming care.

Privately funded gender-affirming hormone treatment and breast surgery are offered by a very small number of physicians and surgeons in Norway. Gender-affirming facial surgery and privately funded genital surgery can only be obtained abroad.

Norwegian authorities are obliged to provide equal access to adequate transgender-specific health care to those who need it [88–90]. To improve access to and quality of health care, more research is needed on this heterogenous population.

### Aim

The aim of the current study was to describe a broad nationwide sample of Norwegian transgender people and investigate differences between gender identity groups within the sample, in terms of sociodemographic characteristics, transition pathways, gender-affirming health care needs, experiences with care, and health and wellbeing.

## Methods

### Study design and recruitment

This study use data from TransNor, an anonymous online survey among transgender people in Norway, conducted from June 7th to September 3rd, 2023. Inclusion criteria were residing in Norway, being 16-year-old or older, and having a gender identity different from sex assigned at birth.

**Fig. 1:**
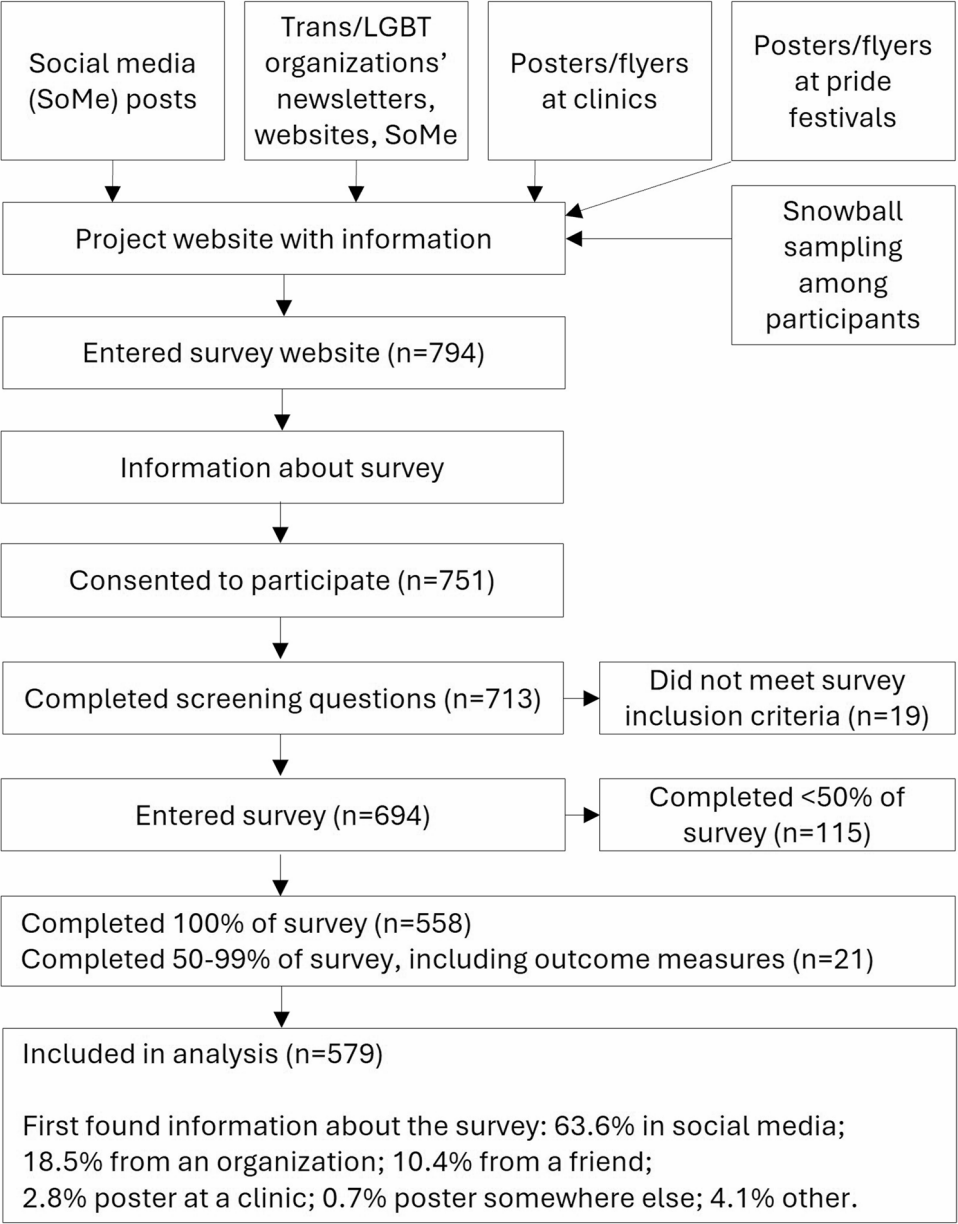
Flow chart of recruitment strategies and survey completion rates

A range of recruitment strategies were employed (Fig. 1): posts in relevant social media channels (e.g., “Transgender in Scandinavia”, “Nonbinary and fluid Norway”); advertising through transgender and LGBT organizations; posters at transgender health clinics; posters at three large pride festivals (Oslo, Bergen and Kristiansand); and survey participants were encouraged to tell others about the survey.

Qualtrics was used as the survey tool. Participants could leave and come back within 72 h to complete the survey. The Qualtrics software had a CAPTCHA (completely automated public Turing test to tell computers and humans apart) to inhibit programmed responses.

The project had a transgender community advisory board of three representatives with extensive community participation and accumulated experience-based knowledge beyond their own personal experiences. Advisory board members had a high degree of involvement in general study design, development of the questionnaire, recruitment, and interpretation of findings. They were compensated for the time they worked on the project.

The study questionnaire was developed as an assembly of carefully considered validated instruments, and some items developed specifically for this study. Where Norwegian translations did not already exist, a forwards-backwards translation procedure was used. Only four questions were mandatory to answer. The questionnaire was piloted in terms of receiving extensive feedback from the advisory board and additional five transgender people regarding phrasing of items and response categories, meaningful and respectful introductions, and survey length.

### Measures

#### Sociodemographic characteristics

To ensure participant anonymity and avoid the risk of indirect identification through compilation of variables, only a limited set of carefully considered sociodemographic variables was collected: age (assessed through 5-year-categories and reported as three age categories), having changed legal gender in the national registry (yes/no), size of residence (four categories), highest completed education level (two categories), annual income (three categories), currently in a relationship (no; yes with one person; yes with several persons), having own biological children (yes/no), and currently having care responsibilities for children (yes/no).

#### Gender identity

Based on considerations of power estimates, gender identity was assessed with three response options: man/trans man; woman/trans woman; and nonbinary. The nonbinary group was later stratified into two groups based on assigned sex at birth. It was explained to participants that for statistical purposes, only a limited number of gender identity categories were available. Gender identity was also assessed with an open question and with a continuous measure (not reported in this publication).

#### Gender-affirming health care

Transition-related counselling was assessed by two questions: have you ever wanted counselling or psychotherapy for your gender identity or transition (yes/no), and have you ever had such counselling (yes/no). Among those having obtained counselling, perceived therapist knowledge about transgender issues was assessed by a 4-point scale from “a lot” to “nothing”, or “I don’t know”, and perceived therapist supportiveness of transgender needs was assessed by a 5-point scale from “very supportive” to “very unsupportive” [21].

Gender-affirming medical treatments, i.e., hormone treatment and various types of surgery, were assessed by several questions (Figure A1 in Appendix A) and later combined into four options for each treatment type: obtained; unmet need; don’t know if they want; don’t want. Participants who had obtained hormones reported their current use of prescription and non-prescription sources. Participants who had obtained hormones or surgery indicated their outcome satisfaction on a 5-point scale from “very satisfied” to “very dissatisfied” and indicated the extent treatment had been obtained through state-funded public health care (all; most; some; none). Participants who had unmet needs for a treatment selected from a list of reasons for not yet having obtained the treatment.

A small number (*n* = 15) had obtained only “other” surgery (Figure A1), and because the type of procedure was unknown, these were excluded from the “obtained surgery” group (Table A6 and Table A7).

#### Transition progress

Participants’ transition progress was assessed by asking participants to rate on a horizontal visual 5-point scale how far they had come in a process of transition or change, from 1 (“I have thought about it, but not made any changes”) to 5 (“I have made most of the changes I want”). This was a slightly modified version of the approach developed by Budge and colleagues [91]. The score was analyzed as a continuous scale variable.

A small number (*n* = 11) responded that they did not want to make any changes, and a small number (*n* = 29) responded that they didn’t know, and these were excluded from analysis of transition progress.

#### General health

General health was assessed by an item with response options ranging from 1 (very good) to 5 (very bad) [92]. Responses were reported as three categories.

#### Life satisfaction

Life satisfaction was assessed by the Satisfaction With Life Scale (SWLS), a 5-item scale designed to measure global cognitive judgments of one’s life satisfaction, and not related constructs such as positive affect or loneliness [93]. Participants indicated to what extent they agreed with each item using a 7-point scale from 1 (strongly disagree) to 7 (strongly agree). The values of all items were summed, and the total score was analyzed as three categories: dissatisfied (5–19), neutral (20–25) and satisfied (26–35) [94]. The Cronbach’s alpha of the SWLS in the current study was 0.891.

#### Mental distress

Mental distress was assessed by the 10-item version of the Hopkins Symptom Checklist (HSCL-10). Participants indicated to what extent they had experienced symptoms of anxiety and depression during the last week, using a 4-point scale from 1 (not affected at all) to 4 (affected a lot). The values of all items were averaged, and the total score was analyzed dichotomously with a score of 1.85 as the clinical cut-off value (i.e., a predictor of a diagnosable mental disorder as assessed by clinical interview) [95]. The Cronbach’s alpha of the HSCL-10 in the current study was 0.895.

#### Social anxiety symptoms

Social anxiety symptoms were assessed by the shortened version of the Social Phobia Inventory (Mini-SPIN), a 3-item scale developed as a brief screening instrument for social anxiety disorder [96]. Participants indicated to what extent they had experienced typical symptoms of social anxiety during the last week, using a 5-point scale from 0 (not affected at all) to 4 (affected a lot). The values of all items were summed, and the total score was analyzed dichotomously with a score of 6 as the clinical cut-off value [96]. The Cronbach’s alpha of the Mini-SPIN in the current study was 0.822.

#### Suicide attempts

Suicide attempts were assessed by both lifetime occurrence and during the last 12 months (yes/no).

### Statistical analysis

SPSS version 29.0 was used for statistical analysis. Chi-Square Test for Independence was used, with Cramer’s *v* as estimate of effect size (0.06 is considered a small effect, 0.17 a medium effect and 0.29 a large effect) [97]. Z-tests with Bonferroni correction were used for post hoc analyses, reported as subscript letters in table rows where each subscript letter denotes a subset of groups that do not differ significantly from each other at the 0.05 level. One-way analysis of variance (ANOVA) was used, with eta squared (η^2^) as estimate of effect size (0.01 is a small effect, 0.06 a medium effect and 0.14 a large effect) [97].

## Results

### Sociodemographic characteristics

Of the total sample, 37.0% identified as man or trans man, 33.7% as woman or trans woman, and 29.4% as nonbinary. Among participants assigned female at birth (AFAB), 38.9% identified as nonbinary, and among participants assigned male at birth (AMAB), 14.8% identified as nonbinary. Trans men and trans women were more likely to have changed legal gender, compared to nonbinary people (Table 1). The majority (74.8%) of the total sample were below 35 years of age, and trans men and nonbinary AFAB people were younger, had lower income and were less likely to have own biological children, compared to trans women and nonbinary AMAB people.

**Table 1:** Sociodemographic characteristics by gender identity group

	Total		Man/trans man		Woman/trans woman		Nonbinary AFAB		Nonbinary AMAB		*p*-value
	n	(%)	n	(%)	n	(%)	n	(%)	n	(%)	
Assigned sex at birth											
AFAB	350	(60.4)	214				136				
AMAB	229	(39.6)			195				34		
Have changed legal gender	275	(47.6)	135	(63.1)^a^	118	(60.8)^a^	19	(14.0)^b^	3	(8.8)^b^	<.001
Age											
16–24	202	(34.9)	93	(43.5)^a^	45	(23.1)^b^	58	(42.6)^a^	6	(17.6)^b^	<.001
25–34	231	(39.9)	85	(39.7)	79	(40.5)	52	(38.2)	15	(44.1)	.931
>34	146	(25.2)	36	(16.8)^a^	71	(36.4)^b^	26	(19.1)^a,c^	13	(38.2)^b,c^	<.001
Urban-rural											
Oslo (capital; 709k)	142	(24.9)	49	(23.4)	52	(26.7)	33	(24.8)	8	(23.5)	.897
Large city (>100k)	159	(27.8)	49	(23.4)	55	(28.2)	44	(33.1)	11	(32.4)	.242
Medium city (20k-100k)	162	(28.4)	65	(31.1)	53	(27.2)	34	(25.6)	10	(29.4)	.697
Rural area	108	(18.9)	46	(22.0)	35	(17.9)	22	(16.5)	5	(14.7)	.512
Education level											
Primary or high school	284	(49.8)	114	(54.3)	89	(46.1)	68	(51.1)	13	(38.2)	.199
College or university	286	(50.2)	96	(45.7)	104	(53.9)	65	(48.9)	21	(61.8)	.199
Annual income^1^											
NOK <300k	321	(60.2)	124	(66.0)^a^	94	(49.2)^b^	86	(71.7)^a^	17	(50.0)^a,b^	<.001
NOK 300k-500k	91	(17.1)	35	(18.6)	35	(18.3)	18	(15.0)	3	(8.8)	.471
NOK >500k	121	(22.7)	29	(15.4)^a^	62	(32.5)^b^	16	(13.3)^a^	14	(41.2)^b^	<.001
In a long-term relationship	261	(46.2)	92	(44.2)	93	(48.4)	58	(44.3)	18	(52.9)	.676
Biological children	69	(12.2)	9	(4.4)^a^	39	(20.2)^b^	12	(9.1)^a^	9	(26.5)^b^	<.001
Care resp. for children	65	(11.4)	14	(6.7)^a^	26	(13.3)^a,b^	15	(11.4)^a,b^	10	(29.4)^b^	.001

Each subscript letter denotes a subset of groups that do not differ significantly from each other at the .05 level

^1^Median annual income in the general population of Norway was NOK 572k in 2022

### Transition progress

Of the total sample, 82.6% were currently living as their self-identified gender always or almost always, and this was more common among transmen (93.5%), followed by transwomen (80.5%), nonbinary AFAB people (75.7%) and nonbinary AMAB people (52.9%) (*p* <.001) (Table A1). Similarly, the groups differed significantly in their transition progress. The proportion who had made most of the changes they wanted (socially, medically, or other) was higher among transmen (35.4%), followed by trans women (17.6%), nonbinary AFAB people (16.1%) and nonbinary AMAB people (0.0%) (*p* <.001).

**Fig. 2:**
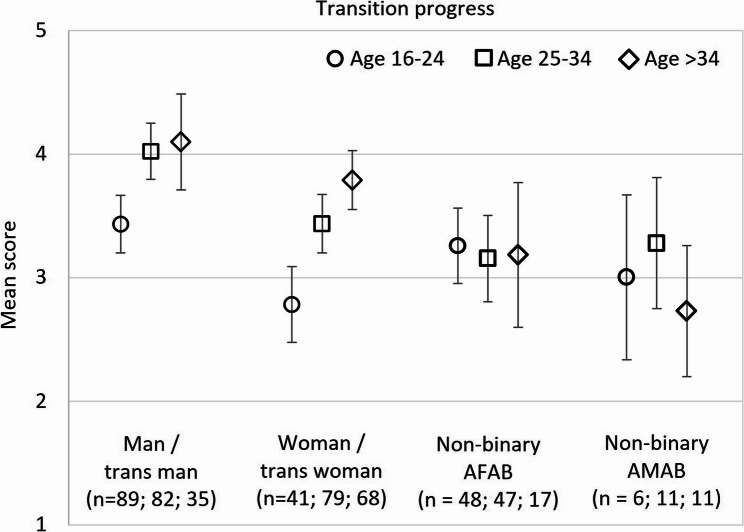
Mean score on the transition progress scale (range 1–5) by gender identity group, stratified by age. 95% CI

Older participants have had more time to make changes and might have moved further in their transition process than younger participants. Therefore, we stratified the total sample by three age groups (Fig. 2 and Table A2). Higher age was associated with higher transition progress only among trans men (*p* <.001; η^2^ = 0.07) and trans women (*p* <.001; η^2^ = 0.12), while among nonbinary people, age was not significantly associated with transition progress.

### Gender-affirming health care

The gender identity groups differed significantly in relation to gender-affirming health care, and effect sizes indicated large differences between the groups (Table 2). Having obtained gender-affirming health care was generally more common among trans men and trans women, as compared to nonbinary people. Unmet treatment needs for all different types of treatments were observed in every gender identity group. The proportion of respondents that did not want or did not know if they wanted a specific treatment differed among treatments.

**Table 2:** Gender-affirming health care by gender identity group

	Total		Man/trans man		Woman/trans woman		Nonbinary AFAB		Nonbinary AMAB		*p*-value	Cramer’s *v *^1^
	n	(%)	n	(%)	n	(%)	n	(%)	n	(%)		
Transition-related counselling												
Wanted and obtained	281	(49.6)	119	(57.2)^a^	108	(56.0)^a^	39	(29.3)^b^	15	(45.5)^a,b^	<.001	.23
Wanted but unmet need	161	(28.4)	40	(19.2)^a^	41	(21.2)^a^	69	(51.9)^b^	11	(33.3)^a,b^	<.001	.30
Did not want, but had	57	(10.1)	32	(15.4)^a^	20	(10.4)^a,b^	4	(3.0)^b^	1	(3.0)^a,b^	.001	.17
Did not want, did not have	68	(12.0)	17	(8.2)	24	(12.4)	21	(15.8)	6	(18.2)	.115	.10
Hormones												
Obtained	341	(59.1)	158	(73.8)^a^	135	(69.9)^a^	36	(26.5)^b^	12	(35.3)^b^	<.001	.41
Unmet need	135	(23.4)	48	(22.4)	54	(28.0)	27	(19.9)	6	(17.6)	.267	.08
Don’t know if they want	74	(12.8)	7	(3.3)^a^	1	(0.5)^a^	55	(40.4)^b^	11	(32.4)^b^	<.001	.51
Don’t want	27	(4.7)	1	(0.5)^a^	3	(1.6)^a^	18	(13.2)^b^	5	(14.7)^b^	<.001	.27
Chest/breast surgery												
Obtained	164	(28.4)	111	(51.9)^a^	23	(11.8)^b,c^	30	(22.4)^c^	0	(0.0)^b^	<.001	.42
Unmet need	237	(41.1)	96	(44.9)^a^	77	(39.5)^a^	59	(44.0)^a^	5	(14.7)^b^	.008	.14
Don’t know if they want	104	(18.0)	6	(2.8)^a^	54	(27.7)^b^	31	(23.1)^b^	13	(38.2)^b^	<.001	.32
Don’t want	72	(12.5)	1	(0.5)^a^	41	(21.0)^b^	14	(10.4)^b^	16	(47.1)^c^	<.001	.37
Internal genital surgery^2^												
Obtained	93	(16.2)	49	(23.1)^a^	30	(15.5)^a,b^	14	(10.4)^b^	0	(0.0)^c^	<.001	.17
Unmet need	230	(40.1)	72	(34.0)^a^	113	(58.2)^b^	37	(27.6)^a^	8	(23.5)^a^	<.001	.27
Don’t know if they want	162	(28.2)	67	(31.6)^a^	32	(16.5)^b^	50	(37.3)^a^	13	(38.2)^a^	<.001	.19
Don’t want	89	(15.5)	24	(11.3)^a^	19	(9.8)^a^	33	(24.6)^b^	13	(38.2)^b^	<.001	.23
External genital surgery^3^												
Obtained	44	(7.7)	13	(6.2)^a^	28	(14.4)^b^	3	(2.2)^a^	0	(0.0)^a,b^	<.001	.19
Unmet need	162	(28.3)	61	(29.0)^a^	90	(46.2)^b^	10	(7.5)^c^	1	(2.9)^c^	<.001	.35
Don't know if they want	192	(33.5)	90	(42.9)^a^	47	(24.1)^b^	40	(29.9)^a,b^	15	(44.1)^a,b^	<.001	.18
Don’t want	175	(30.5)	46	(21.9)^a^	30	(15.4)^a^	81	(60.4)^b^	18	(52.9)^b^	<.001	.40

Each subscript letter denotes a subset of groups that do not differ significantly from each other at the .05 level

^1^Cramer’s *v* effect size: .06 is a small effect, .17 a medium effect and .29 a large effect

^2^Removal of testicles, ovaries and/or uterus

^3^Vaginoplasty, metoidioplasty or phalloplasty

Among trans women and nonbinary AMAB people, a proportion reported unmet needs for facial feminization surgery (44.1% and 29.4%), tracheal shave (28.7% and 14.7%) and voice feminization surgery (27.2% and 8.8%); only a small proportion of trans women had obtained such surgeries (5.6%, 5.1% and 1.5%) (Table A3).

Among participants who had obtained transition-related counselling, 39.5% perceived the therapist as having a high level of knowledge about transgender topics, and 76.3% perceived the therapist as supportive of their needs as a transgender person, and gender identity groups did not differ significantly (Table A4). Those who had counselling despite not wanting it did to a greater extent perceive the therapist as unsupportive, as compared to those who wanted the counselling (24.6% vs. 11.1%, *p* <.01, Table A5).

Participants who had obtained gender-affirming medical treatments generally reported high satisfaction with treatment outcomes (Table 3).

More than half (58.4%) of the total sample had experienced discrimination related to their gender identity or expression when accessing health care and therefore had difficulties getting medical or mental health treatment (transition-related or other); gender identity groups did not differ significantly in their experience of discrimination (not shown in table). Barriers to care were evident both among people who had obtained gender-affirming treatments and among people who had unmet needs (Table 3). Among those having obtained hormones, one third had obtained it entirely through private funding, and this was more common among nonbinary people compared to trans men. Among those having obtained surgery, half had obtained it entirely through private funding. Among participants with unmet treatment needs, almost half could not afford hormones, more than half could not afford surgery, and one out of five had consulted a health professional but was refused treatment. Furthermore, compared to trans men and trans women with unmet needs, nonbinary people with unmet needs were less likely to be under assessment (i.e., having sought a medical/psychological evaluation of eligibility for treatment), less likely to be on a waiting list to obtain surgery (i.e., having been approved as eligible for treatment), and more likely to have not sought surgery because of fear of negative reactions from others. Currently obtaining hormones from non-prescription sources and having an unmet need for surgery that is not available in Norway were more common for feminizing than masculinizing treatments.

**Table 3:** Experiences with gender-affirming treatments, and reasons for not yet having obtained treatments, by gender identity group^1^

	Total		Man/trans man		Woman/trans woman		Nonbinary AFAB		Nonbinary AMAB		*p*-value
	n	(%)	n	(%)	n	(%)	n	(%)	n	(%)	
Proportions among those who had obtained hormones (n=341)											
Very or slightly satisfied with hormone treatment outcome	319	(93.3)	155	(98.1)^a^	121	(89.0)^b^	33	(91.7)^a,b^	10	(83.3)^b^	.008
Obtained hormones entirely through private funding	111	(32.5)	37	(23.4)^a^	50	(36.8)^a,b^	17	(47.2)^b^	7	(58.3)^b^	.003
Currently obtains hormones with prescription from a doctor	315	(94.6)	153	(97.5)^a^	124	(93.2)^a^	31	(96.9)^a^	7	(63.6)^b^	<.001
Currently obtains hormones from non-prescription sources	49	(14.7)	12	(7.6)^a^	30	(22.6)^b,c^	1	(3.1)^a,c^	6	(54.5)^b^	<.001
Proportions among those who had obtained surgery (n=186)											
Very or slightly satisfied with surgery outcome	178	(95.7)	109	(95.6)	37	(94.9)	32	(97.0)			.907
Obtained surgery entirely through private funding	92	(49.5)	50	(43.9)	22	(56.4)	20	(60.6)			.148
Proportions among those who had unmet needs for hormones (n=135)											
Unable to afford hormone treatment	56	(45.9)	18	(41.9)	20	(42.6)	15	(57.7)	3	(50.0)	.574
Currently under assessment for hormone treatment	73	(58.4)	30	(68.2)^a^	36	(72.0)^a^	5	(20.0)^b^	2	(33.3)^a,b^	<.001
Currently on a waiting list to start hormone treatment	27	(22.1)	11	(25.0)	12	(26.1)	3	(11.5)	1	(16.7)	.489
Consulted a health professional and was refused treatment	27	(22.3)	6	(13.6)	14	(31.1)	5	(19.2)	2	(33.3)	.214
Not obtained hormones because of fear of negative reactions from others	38	(31.4)	15	(34.1)	15	(33.3)	6	(23.1)	2	(33.3)	.784
Proportions among those who had unmet needs for surgery (n=397)											
Unable to afford surgery	260	(65.5)	83	(58.0)^a^	106	(67.1)^a,b^	59	(77.6)^b^	12	(60.0)^a,b^	.030
Currently under assessment for surgery	147	(37.8)	60	(43.2)^a^	70	(44.0)^a^	16	(22.2)^b^	1	(5.3)^b^	<.001
Currently on a waiting list to obtain surgery	97	(25.7)	48	(35.3)^a^	41	(27.5)^a^	7	(9.6)^b^	1	(5.3)^a,b^	<.001
Consulted a health professional and was refused treatment	70	(18.5)	28	(20.4)	29	(19.3)	12	(16.4)	1	(5.3)	.419
Not obtained surgery because of fear of negative reactions from others	82	(21.5)	19	(14.0)^a^	32	(21.1)^a,b^	22	(29.7)^b^	9	(45.0)^b^	.003
The type of surgery needed is not available in Norway	165	(42.7)	49	(36.0)^a^	88	(56.1)^b^	19	(26.0)^a^	9	(45.0)^a,b^	<.001

Each subscript letter denotes a subset of groups that do not differ significantly from each other at the .05 level

^1^Each table row represents a separate variable. Some variables had a small number of missing responses, and the table shows percentages of completed (non-missing) responses. See Table A6, A7, A8 and A9 for complete tables

### Health and wellbeing

Almost half of the participants reported good general health; almost two-thirds reported being dissatisfied with their life; three quarters reported clinical levels of mental distress, and more than one-third reported having attempted suicide (Table 4). Health and wellbeing mostly did not differ significantly between gender identity groups.

**Table 4:** Health and wellbeing by gender identity group

	Total		Man/trans man		Woman/trans woman		Nonbinary AFAB		Nonbinary AMAB		*p*-value
	n	(%)	n	(%)	n	(%)	n	(%)	n	(%)	
General health											
Fairly bad or very bad	138	(23.9)	45	(21.1)	41	(21.0)	44	(32.6)	8	(23.5)	.060
Neither good nor bad	166	(28.8)	60	(28.2)	51	(26.2)	43	(31.9)	12	(35.3)	.568
Fairly good or very good	273	(47.3)	108	(50.7)^a^	103	(52.8)^a^	48	(35.6)^b^	14	(41.2)^a,b^	.010
Life satisfaction											
Dissatisfied	354	(62.3)	120	(57.1)	124	(64.2)	86	(65.6)	24	(70.6)	.230
Neutral	143	(25.2)	58	(27.6)	47	(24.4)	30	(22.9)	8	(23.5)	.767
Satisfied	71	(12.5)	32	(15.2)	22	(11.4)	15	(11.5)	2	(5.9)	.370
Mental distress above clinical cut-off	431	(74.8)	162	(76.1)	141	(72.3)	104	(77.6)	24	(70.6)	.636
Social anxiety above clinical cut-off	302	(53.1)	119	(56.4)^a^	86	(44.8)^a^	78	(59.1)^a^	19	(55.9)^a^	.041
Suicide attempt											
Ever	211	(36.7)	95	(44.6)^a^	66	(34.0)^a^	42	(31.3)^a^	8	(23.5)^a^	.015
Last 12 months	27	(4.7)	14	(6.6)	9	(4.6)	3	(2.3)	1	(2.9)	.298

Each subscript letter denotes a subset of groups that do not differ significantly from each other at the .05 level

## Discussion

The aim of the study was to describe a broad national sample of Norwegian transgender people and investigate differences between gender identity groups within the sample. Roughly one third (37.0%) of the sample identified as man or trans man, one third (33.7%) as woman or trans woman, and one third (29.4%) as nonbinary, the latter being more common among AFAB (38.0%) than AMAB (14.8%) people. Having obtained gender-affirming health care was more common among trans men and trans women as compared to nonbinary people. Unmet needs for treatments were observed in every gender identity group. Having obtained treatment entirely through private funding was reported by one third (32.5%) of those who had obtained hormones and half (49.5%) of those who had obtained surgery. Approximately half (47.3%) of the total sample reported good general health, almost two-thirds (62.3%) reported low levels of life satisfaction, almost three quarters (74.8%) reported mental distress above clinical cut-off, and more than one third (36.7%) had ever attempted suicide.

### Gender-affirming health care

Participants in our study generally reported being satisfied with the outcomes of gender-affirming hormonal and surgical treatments. This is in line with previous research [21, 48, 98].

As elaborated in the WPATH international standards for transgender health care (SOC8), treatment needs cannot be predicted for any individual based solely on their gender identity [32]. This was evident in the present study. We investigated five different types of gender-affirming health care: counselling, hormone treatment, chest/breast surgery, and internal and external genital surgery. Results clearly indicated differences in health care needs, not only between gender identity groups, but also within gender identity groups. Overall, the gender identity groups were internally heterogenous regarding treatment needs, i.e., a considerable proportion in every gender identity group did not want or did not know if they wanted specific treatments. Notable exceptions were that most trans men and trans women expressed a need for hormones, and most trans men expressed a need for chest surgery. One implication from these findings is that provision of gender-affirming health care should be tailored to individual treatment needs, and not solely be based on gender identity group membership.

Although the current study did not assess why participants did not know if they wanted specific treatments, several aspects may explain this. Lack of information about available treatment options, particularly for nonbinary people, could make treatment decisions more difficult [78]. More importantly, the need for a specific treatment, e.g., surgery, may be dependent on the outcome of a different treatment, e.g., hormones. Some may want to assess the bodily and psychological effects of hormone treatment, before making decisions about surgery. Decisions as well as uncertainty may also be dependent on other factors such as sexual needs, reproductive needs, relationship status and other life circumstances, and transgender people may consider benefits and risks related to treatment outcomes as well as social consequences of medical transition [99]. Thus, providers of health care must be attentive to the individual needs and life situations of patients.

Among participants who had obtained counselling or psychotherapy for their gender identity or transition, 39.5% perceived the therapist as having a high level of knowledge about transgender topics. Although the current study did not assess therapist level of knowledge directly, the results indicate a need for increased availability of therapists with adequate training in transgender topics. In Norway, this should be aimed for not only through regional or local transgender-specific health centers that are being established but also through public mental health services and school health services. Transition-related counselling can include affirmative exploration of gender identity and treatment needs, psychoeducation on the impact of minority stress and the internalization of prejudice, as well as promoting resilience such as identity pride and building supportive social networks.

### Differences between trans men, trans women, and nonbinary people

Trans women, despite being older than trans men, reported lower transition progress and had to a lesser extent made the changes they wanted. For some trans women, feminization of body and gender expression might require a larger number of interventions or transition steps, such as permanent hair removal, voice training, facial feminization surgery and other surgical interventions, which may be less accessible, or simply too costly due to lack of coverage from the state-funded health care system. A growing body of research indicates that facial feminization surgeries are associated with improved quality of life and psychosocial outcomes [100–102].

Compared to trans men and trans women, nonbinary people reported lower transition progress and were to a lesser extent living as their self-identified gender. Nonbinary people may have a “less linear and more flexible transition pathway that may start at later stages in their lives and have a less specific end point” [31]. Nonbinary people may to a lesser extent desire gender-affirming medical interventions [103–105], and some research indicates that they to a lesser extent experience body discomfort [99, 106, 107], or their experiences are more diverse and differ from a traditional binary concept of gender dysphoria [108, 109]. However, some social and medical transition steps desired by nonbinary people might be unavailable to them. Nonbinary people may need a change of legal gender to affirm their gender identity, but the Norwegian government currently do not allow a third legal gender option in official identity documents [110], despite calls from The Norwegian Directorate for Children, Youth and Family Affairs for increased legal recognition of nonbinary people [111]. Traditionally, medical gender transition was based on a binary understanding of gender. Some nonbinary people may present with other treatment needs, such as tailored hormone treatment with lower dosage or duration [112–114] or masculinizing chest surgery to obtain a non-flat, androgynous-appearing chest instead of a flatter, masculine-appearing chest typically desired by trans men [115, 116]. Fear of negative reactions from others was a more common reason for not obtaining surgery among nonbinary people compared to trans men.

### Barriers to care

Gender-affirming medical treatments are based on decades of clinical experience and research and has been found effective at reducing gender incongruence and gender dysphoria, and thus, are generally considered as medically necessary health care for transgender people in need of it [32]. This view is sometimes challenged, such as in discussions on appropriate care for transgender adolescents [117], nonbinary people, or for specific procedures such as facial feminization surgery [118]. In Norway, medically necessary health care is provided through the state-funded public health care system, and residents do not need private funding of such health care. Despite the existence of publicly funded gender-affirming care, a high proportion of participants in the sample, of which many were young and had low income, had obtained gender-affirming hormone treatment or surgery entirely through private funding. Among participants with unmet treatment needs, a high proportion reported that they could not afford treatment. This is particularly alarming given the high rates of mental distress reported by participants in our study, and evidence that gender-affirming health care is associated with reduced mental distress [36, 37, 40, 44, 45]. The findings indicate a need for increased access to medically necessary gender-affirming health care through the state-funded public health care system in Norway.

The current study did not directly assess reasons why participants chose privately funded care despite the existence of a publicly funded clinic. Multiple explanations can be hypothesized, such as long waiting times [61, 86], the one-year minimum assessment period currently required by the public clinic [84], concern that the psychiatric evaluation or readiness assessment could postpone or exclude access to treatment [119–121], the perception that providers lack recognition of gender diversity [61], experienced or anticipated exclusion of nonbinary people from care [61, 71, 78], and experienced or anticipated non-provision of individualized care such as tailored hormone treatment.

Some participants may have obtained treatment through private funding after being refused treatment from public health care. A limitation of the current study was that treatment refusal was only assessed among participants with unmet treatment needs, of which one out of five (18.5%−22.3%) had consulted a health professional and was refused treatment. Reasons for treatment refusals could be multitude and were not assessed in the current study. Important in this context, the WPATH Standards of Care have over time evolved from a gatekeeping model towards an informed consent model for gender-affirming care, with a shift in ethical considerations from “do no harm” to the core principle of patient autonomy, where expressed patient needs for gender-affirming care are given more weight [120].

Compared to trans men, nonbinary people were more likely to have obtained hormone treatment entirely through private funding. Compared to trans men and trans women with unmet needs for hormones or surgery, nonbinary people with unmet needs were less likely to be under assessment and less likely to be on a waiting list to obtain surgery. This may in part be due to public health care policies which implicitly or explicitly exclude nonbinary people from accessing gender-affirming medical treatments [61]. Some nonbinary people might feel pressure to present as more binary or conceal their nonbinary identity in clinical settings [78]. To reduce barriers to care, policies of state-funded health care should align with the WPATH Standards of Care (SOC8) which recommend that nonbinary people are provided individualized assessment and treatment that affirms their experience of gender [32].

Nonbinary AFAB people were less likely to have obtained transition-related counselling, and more likely to have unmet needs for counselling, as compared to trans men and trans women. Nonbinary people may avoid disclosing their gender identity because they anticipate or experience therapists and primary health care providers to lack specific knowledge about nonbinary identities and health care needs [122].

Having had transition-related counselling despite not wanting it was reported by 10.1% of the total sample, and these were less likely to perceive their therapist as supportive compared to those who wanted the counselling. Counselling or psychotherapy as a mandatory prerequisite for gender-affirming medical treatments is not recommended in the WPATH Standards of Care (SOC8) [32]. It can be a harmful barrier to care for those who do not need counselling or lack access to therapists with adequate training in transgender issues.

Of the total sample, 58.4% had experienced discrimination related to their gender identity or expression when accessing health care (transition-related or other). To prevent discrimination in health care settings, and make transgender people feel safe disclosing their transgender status, health service providers in general need up-to-date knowledge about transgender identities and health care needs [56, 123].

### Health and wellbeing

In the current study of the transgender population in Norway, 36.7% of the total sample reported having ever attempted suicide. This is nearly 12 times compared to the general population of Norway (3.1%) [124]. In addition, 74.8% reported levels of mental distress above clinical cut-off, which is an almost fourfold prevalence compared to the general population (20%) [125]. Satisfaction with life was reported by 12.5%, which is lower than the general population (56.2%; results based on first author’s analysis of the Statistics Norway Quality of Life Survey 2023 dataset [126]). In terms of overall physical and mental health, 47.3% assessed their general health as very or fairly good, which is lower than the general population (68.0%) [125].

The health status of the total sample in the current study were generally in line with findings from previous studies on the transgender population in Norway. The 36.7% prevalence of suicide attempts resemble the 30–34% found in a nationwide transgender sample [6] but was higher than the 21.4–23.2% found in a sample of transgender students [13] and higher than the 27.0% found in a regional sample of transgender people [71]. The 74.8% prevalence of clinical mental distress found in the current study resembled the 71.4–74.6% reported by transgender students [13] but was higher than the 63.0–67.0% found in a nationwide transgender sample [6]. And the 47.3% proportion having good general health was lower than 56.0–57.0% found in a nationwide [6] and 56.0% found in a regional transgender sample [71].

It is important to note that the current study also documented positive outcomes and indicators of resilience. Approximately half (47.3%) of the study sample reported good general health, half (50.2%) had completed college- or university-level education, half (46.2%) were in a long-term relationship, and 11.4% had care responsibilities of children. The majority (82.6%) were living as their self-identified gender, and most participants had completed at least some of their desired transition steps. Among those having obtained gender-affirming medical treatments, a very large proportion (93.3% and 95.7%) were satisfied with the outcomes of hormone treatment and surgery. Obtaining gender-affirmation despite prejudice and structural barriers indicates agency and strength in the pursuit of good health and life satisfaction.

There were few significant differences between the gender identity groups on health and wellbeing. Previous research in various countries has been mixed on this issue, with some studies indicating better health and others worse health among nonbinary as compared to binary transgender people [127]. A meta-analysis of 21 studies from six countries found that nonbinary youth reported poorer general mental health than binary transgender youth, while no significant differences was found on depressive symptoms, anxiety, or lifetime or past year suicide attempts, with moderate heterogeneity among studies [128]. Transgender people face challenges that are shared by gender identity groups within this population, as well as challenges that are unique or more pronounced for specific gender identity groups, which may vary across culture and social context.

The study sample comprised participants that were currently at various stages of transition, i.e., pre-transition, mid-transition, and post-transition. Gender minority stress can be experienced regardless of transition status. Lifetime and past year suicide attempts as well as mental distress reported by participants can be influenced by factors unrelated to minority stress and gender transition. However, several empirical studies and reviews clearly indicate that minority stressors such as discrimination, victimization and non-affirmation of gender identity are positively associated with mental distress and suicidality [24, 25, 29]. Conversely, obtaining medically necessary gender-affirming health care is negatively associated with mental distress and suicidality [37, 43–45]. Thus, having unmet needs for gender-affirming care and experiencing barriers to such care could lead to prolonged mental health problems and reduced quality of life [47, 129–131]. This points to a need for increased access to medically necessary gender-affirming health care through the state-funded public health care system in Norway.

### Strengths and limitations

The relatively large sample size, with a good distribution across gender identity groups within the study population, allowed for detailed descriptive statistics on variables of high relevance for the study population. Using non-probability sampling techniques is considered an appropriate method for recruiting hard-to-reach and stigmatized populations, but it carries a risk of selection bias. We used multiple parallel recruitment strategies to ensure diversity of survey participants. Although a large proportion of participants were of younger ages, the sample had a broad distribution on key variables such as gender identity, sociodemographic characteristics, transition progress, health care needs, treatment status and experiences with heath care. Since we do not know to what extent the sample is representative of the transgender population in Norway, generalizations should be made with caution.

An important strength of the current study was the high level of involvement of a transgender community advisory board in study design and implementation. This contribution strengthened the relevance and precision of research themes, survey questions and interpretation of findings.

Extensive questionnaire piloting regarding aspects such as phrasings, meaningfulness and length likely contributed to high survey participation and completion rates.

## Conclusions

Transgender people in Norway reported high levels of mental distress and suicidality. Despite being young and with low income, a large proportion had obtained gender-affirming medical treatments entirely through private funding. Transgender people in need of gender-affirming health care could benefit from increased access to care through the state-funded public health care system in Norway.

## Supplementary Information

Supplementary Material 1.

## Data Availability

The datasets generated and/or analyzed during the current study will be available in the Sikt public archive after the doctoral project is completed: https://sikt.no/en/tjenester/finn-data/survey-bank.

## Abbreviations

AFAB: Assigned female at birth
AMAB: Assigned male at birth
NOK: Norwegian Krone
U.S.: United States
SWLS: Satisfaction With Life Scale
HSCL: Hopkins Symptom Checklist
SPIN: Social Phobia Inventory
ANOVA: Analysis of variance
SoMe: Social media
CAPTCHA: Completely automated public Turing test to tell computers and humans apart
WPATH: World Professional Association for Transgender Health
SOC8: Standards of Care for the Health of Transgender and Gender Diverse People, Version 8
