# Supplementary material for: Gender-affirming health care needs, barriers to care, and health and wellbeing in a broad nationwide sample of transgender people in Norway
Source: BMC Public Health. 2025 Dec 5;25:4218. doi: 10.1186/s12889-025-25243-1 (PMC12681104; doi:10.1186/s12889-025-25243-1)
Supplement: Supplementary file 1 — Supplementary Material 1. [file 12889_2025_25243_MOESM1_ESM.pdf]

Appendix A

Gender-affirming health care needs, barriers to care, and health and wellbeing in a broad nationwide sample of transgender people in Norway

Bolstad, Silje-Håvard; Anderssen, Norman; Hansen, Bjørge H.; & Meyer, Ilan H.

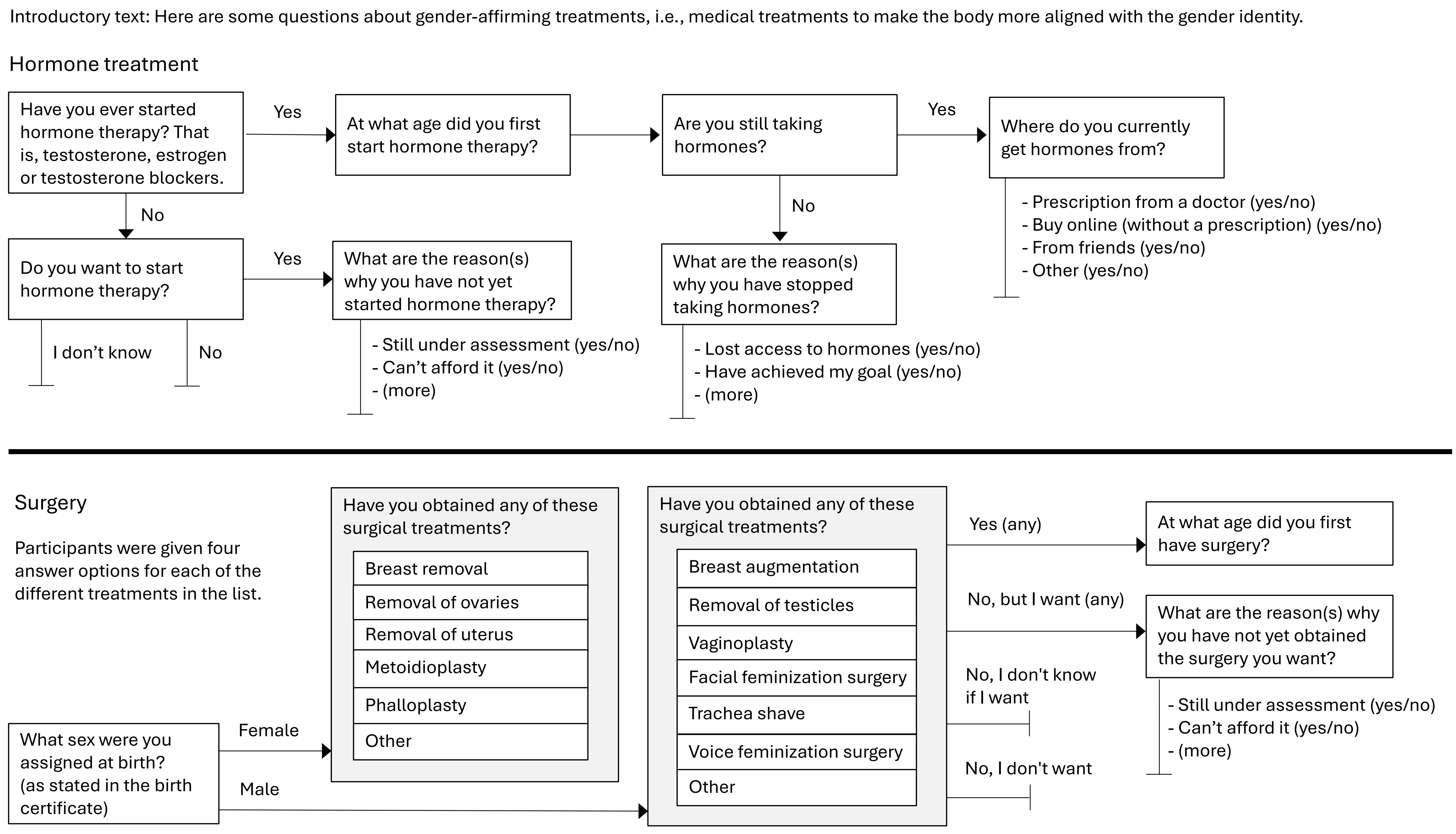

Figure A1: Flow chart of survey questions about gender-affirming medical treatments.

Table A1: Currently living as self-identified gender always or almost always, and transition progress, by gender identity group.

	Total		Man / trans man		Woman / trans woman		Nonbinary AFAB		Nonbinary AMAB		
	n	(%)	n	(%)	n	(%)	n	(%)	n	(%)	*p*-value
Living as self-identified gender always or almost always	478	(82.6)	200	(93.5) [a]	157	(80.5) [b]	103	(75.7) [b,c]	18	(52.9) [c]	**< .001**
Transition progress											
1 I have thought about it, but not made any changes	7	(1.3)	0	(0.0) [a]	4	(2.1) [a,b]	2	(1.8) [a,b]	1	(3.6) [b]	.171
2	114	(21.3)	36	(17.5) [a]	38	(20.2) [a,b]	35	(31.3) [b]	5	(17.9) [a,b]	**.033**
3	150	(28.1)	48	(23.3) [a]	55	(29.3) [a,b]	32	(28.6) [a,b]	15	(53.6) [b]	**.009**
4	139	(26.0)	49	(23.8)	58	(30.9)	25	(22.3)	7	(25.0)	.305
5 I have made most of the changes I want	124	(23.2)	73	(35.4) [a]	33	(17.6) [b]	18	(16.1) [b]	0	(0.0) [b]	**< .001**

Table A2: Mean score on the transition progress scale (range 1-5) by gender identity group, stratified by age group. One-way ANOVA.

	Age 16-24					Age 25-34					Age >34								
				95% CI					95% CI					95% CI					
	n	*M*	*SD*	Lower	Upper	*n*	*M*	*SD*	Lower	Upper	n	*M*	*SD*	Lower	Upper	*df*	*F*	*p*-value	η^2
Man / trans man	89	3.43	1.10	3.20	3.66	82	4.01	1.04	3.78	4.24	35	4.09	1.12	3.70	4.47	2, 203	8.09	**< .001**	.07
Woman / trans woman	41	2.78	.96	2.48	3.08	79	3.43	1.05	3.20	3.66	68	3.78	.97	3.54	4.02	2, 185	12.70	**< .001**	.12
Nonbinary AFAB	48	3.25	1.04	2.95	3.55	47	3.15	1.18	2.80	3.50	17	3.18	1.13	2.59	3.76	2, 109	.10	.904	.00
Nonbinary AMAB	6	3.00	.63	2.34	3.66	11	3.27	.79	2.74	3.80	11	2.73	.79	2.20	3.26	2, 25	1.42	.260	.10

Table A3: Gender-affirming feminizing surgeries by gender identity group.

	Total		Woman / trans woman		Nonbinary AMAB		
	n	(%)	n	(%)	n	(%)	*p*-value
Facial feminization surgery							
Obtained	11	(4.8)	11	(5.6)	0	(0.0)	.156
Unmet need	96	(41.9)	86	(44.1)	10	(29.4)	.109
Don't know if they want	74	(32.3)	64	(32.8)	10	(29.4)	.695
Don’t want	48	(21.0)	34	(17.4) [a]	14	(41.2) [b]	**.002**
Tracheal shave surgery							
Obtained	10	(4.4)	10	(5.1)	0	(0.0)	.177
Unmet need	61	(26.6)	56	(28.7)	5	(14.7)	.088
Don't know if they want	73	(31.9)	61	(31.3)	12	(35.3)	.643
Don’t want	85	(37.1)	68	(34.9)	17	(50.0)	.092
Voice feminization surgery							
Obtained	3	(1.3)	3	(1.5)	0	(0.0)	.467
Unmet need	56	(24.5)	53	(27.2) [a]	3	(8.8) [b]	**.022**
Don't know if they want	79	(34.5)	67	(34.4)	12	(35.3)	.916
Don’t want	91	(39.7)	72	(36.9) [a]	19	(55.9) [b]	**.037**

Table A4. Perceived therapist level of knowledge about transgender topics, and perceived therapist level of support of their needs as a transgender person, by gender identity group. Proportions among participants who had transition-related counselling.

	Total		Man / trans man		Woman / trans woman		Nonbinary AFAB		Nonbinary AMAB		
	n	(%)	n	(%)	n	(%)	n	(%)	n	(%)	*p*-value
Therapist level of knowledge											
Nothing	65	(19.2)	36	(23.8)	21	(16.5)	4	(9.3)	4	(23.5)	.133
A little	81	(24.0)	35	(23.2)	33	(26.0)	11	(25.6)	2	(11.8)	.618
Some	53	(15.7)	21	(13.9)	23	(18.1)	6	(14.0)	3	(17.6)	.784
A lot	130	(38.5)	56	(37.1)	46	(36.2)	20	(46.5)	8	(47.1)	.552
I don't know	9	(2.7)	3	(2.0)	4	(3.1)	2	(4.7)	0	(0.0)	.681
Therapist level of support											
Unsupportive	45	(13.4)	22	(14.7)	13	(10.2)	7	(16.3)	3	(17.6)	.598
Neither supportive nor unsupportive	35	(10.4)	17	(11.3)	14	(11.0)	3	(7.0)	1	(5.9)	.775
Supportive	257	(76.3)	111	(74.0)	100	(78.7)	33	(76.7)	13	(76.5)	.835

Table A5. Perceived therapist level of knowledge about transgender topics, and perceived therapist level of support of their needs as a transgender person, by participants' desire or non-desire for transition-related counselling. Proportions among participants who had transition-related counselling.

	Total		Wanted and obtained		Did not want, but had		
	n	(%)	n	(%)	n	(%)	*p*-value
Therapist level of knowledge							
Nothing	65	(19.3)	53	(18.9)	12	(21.1)	.711
A little	81	(24.0)	67	(23.9)	14	(24.6)	.919
Some	53	(15.7)	42	(15.0)	11	(19.3)	.417
A lot	129	(38.3)	113	(40.4)	16	(28.1)	.082
I don't know	9	(2.7)	5	(1.8) [a]	4	(7.0) [b]	**.026**
Therapist level of support							
Unsupportive	45	(13.4)	31	(11.1) [a]	14	(24.6) [b]	**.007**
Neither supportive nor unsupportive	35	(10.4)	28	(10.0)	7	(12.3)	.613
Supportive	256	(76.2)	220	(78.9) [a]	36	(63.2) [b]	**.011**

Table A6: Satisfaction with treatment outcomes of hormones and surgery, by gender identity group. Proportions among participants who had obtained treatment.

	Total		Man / trans man		Woman / trans woman		Nonbinary AFAB		Nonbinary AMAB		
	n	(%)	n	(%)	n	(%)	n	(%)	n	(%)	*p*-value
Hormones											
Very or slightly dissatisfied	13	(3.8)	3	(1.9)	9	(6.6)	1	(2.8)	0	(0.0)	.165
Neither satisfied nor dissatisfied	10	(2.9)	0	(0.0) [a]	6	(4.4) [b]	2	(5.6) [b]	2	(16.7) [b]	**.002**
Very or slightly satisfied	319	(93.3)	155	(98.1) [a]	121	(89.0) [b]	33	(91.7) [a,b]	10	(83.3) [b]	**.008**
Surgery											
Very or slightly dissatisfied	6	(3.2)	4	(3.5)	2	(5.1)	0	(0.0)			.454
Neither satisfied nor dissatisfied	2	(1.1)	1	(0.9)	0	(0.0)	1	(3.0)			.438
Very or slightly satisfied	178	(95.7)	109	(95.6)	37	(94.9)	32	(97.0)			.907

Table A7: Private funding of gender-affirming medical treatments, by gender identity group. Proportions among those who had obtained treatment.

	Total		Man / trans man		Woman / trans woman		Non-binary AFAB		Non-binary AMAB		
	n	(%)	n	(%)	n	(%)	n	(%)	n	(%)	*p*-value
Hormones											
All	111	(32.5)	37	(23.4) [a]	50	(36.8) [a,b]	17	(47.2) [b]	7	(58.3) [b]	**.003**
Most	43	(12.6)	19	(12.0)	22	(16.2)	0	(0.0)	2	(16.7)	.072
Some	106	(31.0)	57	(36.1)	37	(27.2)	10	(27.8)	2	(16.7)	.246
None	82	(24.0)	45	(28.5)	27	(19.9)	9	(25.0)	1	(8.3)	.199
Surgery											
All	92	(49.5)	50	(43.9)	22	(56.4)	20	(60.6)			.148
Most	15	(8.1)	11	(9.6)	3	(7.7)	1	(3.0)			.467
Some	9	(4.8)	6	(5.3)	1	(2.6)	2	(6.1)			.745
None	70	(37.6)	47	(41.2)	13	(33.3)	10	(30.3)			.429

Table A8: Reasons for not yet having started hormone treatment. Proportions among those who had an unmet need for hormone treatment.

	Total		Man / trans man		Woman / trans woman		Nonbinary AFAB		Nonbinary AMAB		
	n	(%)	n	(%)	n	(%)	n	(%)	n	(%)	*p*-value
Currently under assessment	73	(58.4)	30	(68.2) [a]	36	(72.0) [a]	5	(20.0) [b]	2	(33.3) [a,b]	**< .001**
Looking for a provider	62	(50.0)	17	(39.5)	27	(56.3)	16	(59.3)	2	(33.3)	.238
Can't afford it	56	(45.9)	18	(41.9)	20	(42.6)	15	(57.7)	3	(50.0)	.574
Other	43	(37.7)	16	(38.1)	12	(27.3)	13	(56.5)	2	(40.0)	.138
Fear of negative reactions from others	38	(31.4)	15	(34.1)	15	(33.3)	6	(23.1)	2	(33.3)	.784
Mental health challenge that complicates it	31	(25.6)	14	(32.6)	9	(19.6)	6	(23.1)	2	(33.3)	.523
Consulted a health professional and was refused treatment	27	(22.3)	6	(13.6)	14	(31.1)	5	(19.2)	2	(33.3)	.214
Currently on a waiting list to start treatment	27	(22.1)	11	(25.0)	12	(26.1)	3	(11.5)	1	(16.7)	.489
The type of treatment needed is not available in Norway	18	(14.8)	4	(9.3)	7	(15.2)	5	(18.5)	2	(33.3)	.396
Physical health challenge that complicates it	15	(12.2)	8	(17.8)	4	(8.7)	3	(11.5)	0	(0.0)	.444
The treatment has unwanted side effects	7	(5.7)	1	(2.2)	2	(4.4)	3	(11.5)	1	(16.7)	.250

Table A9: Reasons for not yet having obtained surgery. Proportions among those who had an unmet need for surgery.

	Total		Man / trans man		Woman / trans woman		Nonbinary AFAB		Nonbinary AMAB		
	n	(%)	n	(%)	n	(%)	n	(%)	n	(%)	*p*-value
Can't afford it	260	(65.5)	83	(58.0) [a]	106	(67.1) [a,b]	59	(77.6) [b]	12	(60.0) [a,b]	**.030**
The type of treatment needed is not available in Norway	165	(42.7)	49	(36.0) [a]	88	(56.1) [b]	19	(26.0) [a]	9	(45.0) [a,b]	**< .001**
Currently under assessment	147	(37.8)	60	(43.2) [a]	70	(44.0) [a]	16	(22.2) [b]	1	(5.3) [b]	**< .001**
Other	124	(35.5)	41	(32.3) [a,b]	43	(30.5) [b]	32	(50.0) [a]	8	(47.1) [a,b]	**.030**
Looking for a provider	127	(33.1)	45	(32.6) [a,b]	51	(33.3) [a,b]	30	(40.5) [b]	1	(5.3) [a]	**.036**
Currently on a waiting list to start treatment	97	(25.7)	48	(35.3) [a]	41	(27.5) [a]	7	(9.6) [b]	1	(5.3) [a,b]	**< .001**
Fear of negative reactions from others	82	(21.5)	19	(14.0) [a]	32	(21.1) [a,b]	22	(29.7) [b]	9	(45.0) [b]	**.003**
Consulted a health professional and was refused treatment	70	(18.5)	28	(20.4)	29	(19.3)	12	(16.4)	1	(5.3)	.419
Mental health challenge that complicates it	57	(15.1)	24	(17.8)	14	(9.3)	16	(21.9)	3	(15.8)	.063
Physical health challenge that complicates it	52	(13.7)	26	(19.1)	14	(9.2)	11	(15.3)	1	(5.3)	.064
The treatment has unwanted side effects	42	(11.2)	14	(10.4)	15	(9.9)	9	(12.5)	4	(21.1)	.512
